# Reduction of Morphine During Baking? Response: Commentary: Opium Alkaloids in Harvested and Thermally Processed Poppy Seeds

**DOI:** 10.3389/fchem.2021.692045

**Published:** 2021-05-10

**Authors:** Marcel Kuntz, Patricia Golombek, Dirk W. Lachenmeier

**Affiliations:** Chemisches und Veterinäruntersuchungsamt (CVUA) Karlsruhe, Karlsruhe, Germany

**Keywords:** poppy seeds, *Papaver somniferum*, alkaloids, thermal processing, morphine, codeine, thebaine

The article of Kleinmeier et al. (2021) points out a controversial issue in the scientific literature, namely the potential for reduction of opium alkaloids during thermal processing of poppy seed containing foods. Due to its toxic effects above certain thresholds as well as the potential for confounding drug tests for opiates (Lachenmeier et al., 2010), this question is of significant importance regarding acceptable levels for products intended for baking purposes. While previous literature has generally assumed a considerable decrease of morphine and other alkaloids during baking (Brenneisen and Borner, 1985; Kniel, 2006; Sproll et al., 2006, 2007; Carlin et al., 2020), new data from the U.S. Food and Drug Administration (FDA) (Shetge et al., 2020; also see discussion in Kleinmeier et al., 2021) has shed doubt on this opinion as opiate levels were quite stable when incorporated into a model baked product. Kleinmeier et al. (2021) tried to explain this discrepancy by the possible confounding of previous baking experiments by processing steps including soaking of the poppy seeds, which has been reported to reduce opium alkaloids on the surface of poppy seeds.

As our own previous study (Sproll et al., 2006) was specifically pointed out as being “confounded,” we hereby want to take the chance to rebut this claim, and provide evidence that our study was not confounded by processing steps. The experiment of our baking study was designed as full factorial design with the three factors: ground/unground seeds, long/short cooking time, long/short swelling time according to typical German poppy seed cake baking recipes. The poppy seed was not washed or otherwise treated to reduce the opiate content. The original content in the seed was 270 mg/kg of morphine. The highest recovery in unground seeds was 50%, while the lowest recovery for ground seeds was 16%. From the factors studied, only grinding had a statistically significant influence on the alkaloid content (see full Analysis of Variance results in supporting information for Sproll et al., 2006). In the meantime, we have replicated the results of Sproll et al. (2006), also providing a comparison between raw and baked poppy cake dough (Figure 1).

**Figure 1:**
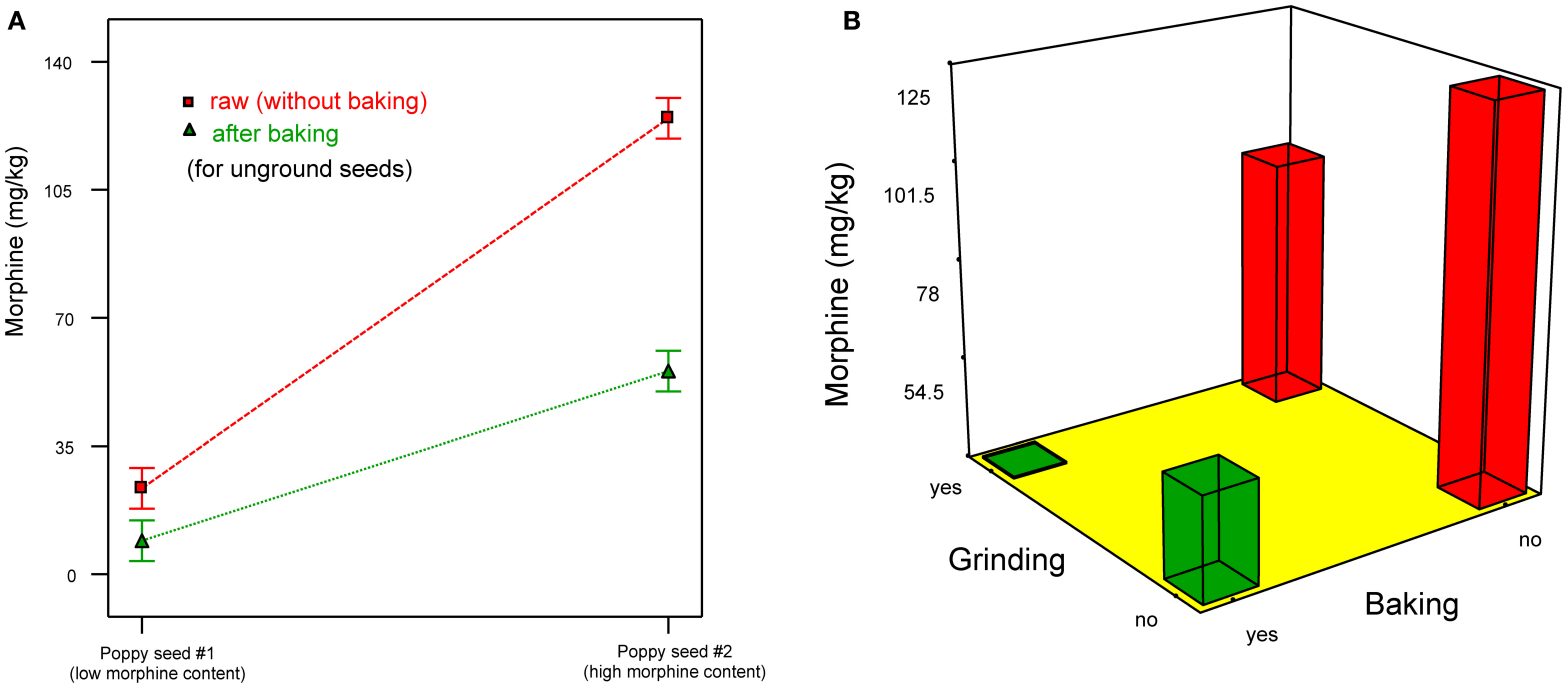
Morphine reduction during poppy cake baking with ground or unground poppy seed [results of full factorial design with 2 morphine levels and two treatments at two levels (with or without grinding and/or baking at 180°C for 20 min), ANOVA *p* < 0.0001, method according to Sproll et al. (2006)]. **(A)** Comparison between raw and baked poppy cake for two different seeds types. **(B)** Influence of baking and grinding for seed type with high morphine content. Both baking and grinding lead to highly significant reduction of morphine. The influence of baking is ~2-times the one of grinding. If both treatments are applied, the reduction is about 70%.

Finally, our results were independently confirmed on a technical scale by the German Baking Agent Institute, which reported tests of fine bakery products containing poppy seeds from commercial bakeries. Production stage controls have shown that the morphine content decreases significantly because of crushing and intensive heating of the poppy seed. In the finished convenience products, morphine was either no longer detectable or only present in comparatively small amounts (Kniel, 2006). It must be also considered that poppy seeds only truly develop their nutty flavor after crushing, blanching and baking, all of which reduce the alkaloid content. Indirect evidence of the feasibility of measures to reduce the opium alkaloid content of food products is provided by the notable reduction of the amounts in commercial products in Germany, since awareness about the problem had been raised in 2005 (Lachenmeier et al., 2010).

For all these reasons, we believe that the literature comprehensively shows that a variety of food processing measures including baking is indeed reducing the opium alkaloid amount (Table 1), and that the result of the U.S. FDA must be considered as outlier, possibly to be explained by the model experiment leading to comparably low interior temperatures inside the muffin (Kleinmeier et al., 2021).

**Table 1:** Changes in morphine content of poppy seed foods during processing according to literature data.

**Food processing of poppy seeds**	**Decrease of morphine level**	**References**
Washing with slightly acidic water	40%	Bjerver et al., 1982
Soaking with water (5 min)	46%	Lo and Chua, 1992
Washing with hot water (2 min)	73 ± 13%	Sproll et al., 2007
Grinding with laboratory mill	34 ± 5%	Sproll et al., 2006
Grinding with commercial poppy mill	25 ± 15%	Sproll et al., 2006
Cake making (180°C, 20 min)	50–84%	Sproll et al., 2006
Bun making (no temperature/time specified)	90%	Brenneisen and Borner, 1985
Bun making (220°C, 20 min)	80–90%	Sproll et al., 2006
Commercial production of baking mixes	100%	Kniel, 2006
Cake making (untreated seeds, 180°C, 20 min)	55–61%	This study (Figure 1)
Cake making (ground seeds, 180°C, 20 min)	70–75%	This study (Figure 1)
Baking of muffin (200°C, 16 min)	No effect	Shetge et al., 2020
Thermal treatment (200°C, 30-40 min)	50%	Shetge et al., 2020
Water washing	50–80%	Shetge et al., 2020
Bread roll (190°C, 25 min)	99–100%	Carlin et al., 2020
Heating, no matrix (180°C, 15 min)	49–100%	Carlin et al., 2020

For the regulatory or toxicological assessment of poppy seeds and poppy seed-containing products, it has therefore to be taken into account that during the preparation of poppy seeds (grinding, baking, and heating) considerable amounts can be eliminated depending on various enzymatic (phenol oxidases) and non-enzymatic effects (Sproll et al., 2007). It must be reiterated from our 2006 study, that the same effects can also occur during laboratory analysis of poppy seeds so that alkaloid-reducing steps such as grinding or heating should be avoided at this stage, which would lead to incorrect, non-reproducible results (Sproll et al., 2006).

In a recent review, Casado-Hidalgo et al. (2021) discussed the current and future perspectives of opium alkaloids in food. They concluded that there are several ways of action to control the levels of opium alkaloids in food products. For example, maximum limits in seeds or foods, selection of certain varieties of poppy plants with seeds for food purposes, good harvesting practices to minimize contamination and good processing practices to minimize the concentration of opium alkaloids. The selection of almost alkaloid-free varieties may be specifically promising to avoid the problem without need for further process controls (Kuntz et al., 2019).

Poppy seed buns are unlikely to pose a hazard to consumers because of the small amounts consumed and the extensive degradation of morphine through the baking process, assuming moderate amounts of morphine in the poppy seed. In Germany, poppy seed cake is likely to be the main source of poppy seed consumption. Nowadays, bakeries rarely make their own poppy seed filling in the cake-making process. Almost exclusively convenience poppy seed fillings are used, which according to the above mentioned studies contain only traces of opiates. Poppy seed cakes from bakeries are thus unlikely to represent a source of danger. However, the morphine content in poppy seed for direct delivery to the consumer should be reduced to the lowest level technologically possible.

Besides the morphine content in food products, attention should be paid to other opium alkaloids in poppy seed, such as thebaine. Up to now, morphine was the main subject of risk assessment of opium alkaloids in poppy seed. However, the limited data concerning the toxic potential of thebaine indicate a higher acute toxicity than morphine (Eisenreich et al., 2020).

We agree that the data currently available is limited and further studies are necessary to enable an extensive hazard characterization. Detailed research on the fate of opium alkaloids during different processing steps in food production as well as research on the toxic potential of different opium alkaloids in poppy seed might be topics for future studies.

## Author Contributions

The idea for the manuscript was conceptualized by DL. MK and PG wrote the first draft of the article. DL revised the article draft. All authors have read and agreed to the published version of the manuscript.

## Conflict of Interest

The authors declare that the research was conducted in the absence of any commercial or financial relationships that could be construed as a potential conflict of interest.
